# Characterizing the community use of an ultralight wheelchair with “on the fly” adjustable seating functions: A pilot study

**DOI:** 10.1371/journal.pone.0173662

**Published:** 2017-03-09

**Authors:** Johanne Mattie, Jaimie Borisoff, William C. Miller, Borna Noureddin

**Affiliations:** 1 MAKE+, British Columbia Institute of Technology, Burnaby, British Columbia, Canada; 2 Department of Occupational Science & Occupational Therapy, UBC Faculty of Medicine, University of British Columbia, Vancouver, British Columbia, Canada; 3 International Collaboration on Repair Discoveries, Vancouver, British Columbia, Canada; 4 Canada Research Chair in Rehabilitation Engineering Design, British Columbia Institute of Technology, Burnaby, British Columbia, Canada; 5 Biomedical Engineering Program, University of British Columbia, Vancouver, British Columbia, Canada; 6 GF Strong Rehabilitation Research Lab, Vancouver, British Columbia, Canada; University of Illinois at Urbana-Champaign, UNITED STATES

## Abstract

An ultralight manual wheelchair that allows users to independently adjust rear seat height and backrest angle during normal everyday usage was recently commercialized. Prior research has been performed on wheelchair tilt, recline, and seat elevation use in the community, however no such research has been done on this new class of manual ultralight wheelchair with “on the fly” adjustments. The objective of this pilot study was to investigate and characterize the use of the two adjustable seating functions available on the Elevation^™^ ultralight dynamic wheelchair during its use in the community. Eight participants had data loggers installed onto their own wheelchair for seven days to measure rear seat height, backrest angle position, occupied sitting time, and distance traveled. Analysis of rear seat height and backrest adjustment data revealed considerable variability in the frequency of use and positions used by participants. There was a wide spread of mean daily rear seat heights among participants, from 34.1 cm to 46.7 cm. Two sub-groups of users were further identified: those who sat habitually at a single typical rear seat height, and those who varied their rear seat height more continuously. Findings also showed that participants used the rear seat height adjustment feature significantly more often than the backrest adjustment feature. This obvious contrast in feature use may indicate that new users of this class of wheelchair may benefit from specific training. While the small sample size and exploratory nature of this study limit the generalizability of our results, our findings offer a first look at how active wheelchairs users are using a new class of ultralight wheelchair with “on the fly” seating adjustments in their communities. Further studies are recommended to better understand the impact of dynamic seating and positioning on activity, participation and quality of life.

## Introduction

The wheelchair is a widely used assistive technology for people with spinal cord injury (SCI) or other mobility impairments. In several studies, wheelchairs have been shown to be an influential factor for people’s independence, ability to perform activities of daily living, and level of participation in the community [1–6]. Unfortunately, many people also feel that their wheelchair poses a greater barrier to participation than their actual mobility impairment, especially if the equipment is poorly matched to their specific activity needs, abilities, and environments of use [2].

Ultralight rigid wheelchairs, or ultralight folding wheelchairs with similar performance specifications, have become a standard provision for people with disabilities who need manual wheelchairs and are active members of the community. For instance, it was found that more than 95% of active veterans who use manual wheelchairs use ultralight wheelchairs [7]. Another study involving people with SCI from six SCI centres in the United States found greater than 80% of the people used ultralight wheelchairs [1]. Current clinical guidelines, especially as they pertain to preservation of upper limb function with chronic wheelchair use, recommend ultralight wheelchairs for active people with disabilities who use manual wheelchairs [8]. Some research points to the beneficial impact that ultralight wheelchairs may have on participation. For instance, people with SCI who used ultralight wheelchairs wheeled significantly more minutes per day compared with those who used lower cost lightweight wheelchairs [1].

Another wheelchair technology that may beneficially impact people with mobility impairments is dynamic seating. The concept of dynamic seating currently refers to either two classes of wheelchair seating. First, it refers to a caregiver’s or user’s ability to easily and quickly (i.e. with client sitting normally in the chair) adjust the seating position during typical wheelchair usage. Manual wheelchairs with this class of dynamic seating include wheelchairs that tilt-in-space or provide backrest recline. Second, dynamic seating may refer to wheelchair seating that moves elastically in response to a user’s movements (e.g. backrests that recline momentarily and absorb energy due to involuntary extensor thrusts). Due to confusion about the use of the term “dynamic seating” we are also using the descriptive term of “on the fly adjustments” to emphasize the difference between these wheelchairs and this new class of ultralight wheelchair described below, that was intended for a different client population.

Another dynamic seating feature, most commonly found in power wheelchairs, is seat elevation (i.e. increasing seat height). According to RESNA (Rehabilitation Engineering Society of North America), seat elevation for power wheelchairs is often medically necessary, and provides benefits associated with participation, such as improving the ability to perform activities of daily living, facilitating transfers, providing psychological benefits by equalizing eye to eye contact with others, and enhancing independence [9]. Other dynamic wheelchair technologies, such as seat tilt, backrest recline, and elevating leg rests, also enable participation [10]. The able-bodied ergonomics field endorses “dynamic sitting” as the only effective way to be productive and functional during extended periods of sitting [11, 12]. RESNA emphatically agrees that dynamic seating should be applied to wheelchair users as well, “since many wheelchair users may not have the same level of dynamic movement as able-bodied” people [10].

Unfortunately, until recently, no dynamic seating or “on the fly” adjustment features were available on the market for active, ultralight wheelchair users. Some ultralight wheelchairs have completely “fixed” frames manufactured to the custom specifications of the user. However, most ultralights offer adjustments to meet an individual users’ general needs (e.g. setting a fixed seat height and back rest position according to the user’s body weight, size, and functional capabilities). These adjustments typically require tools, time, and training. But most significantly, these adjustments cannot be made “on the fly” to allow users to adjust their seated position to match different activities in real-time. The use of dynamic adjustability for ultralight manual wheelchair users has the potential to provide similar benefits noted for power wheelchair users (e.g. enhanced function, comfort, mobility, upper limb injury preservation, etc.).

One ultralight wheelchair that may offer these benefits to manual wheelchair users is the Elevation^™^ wheelchair [13]. The Elevation^™^ wheelchair was designed with two “on the fly” adjustable seating features that enable the user to independently adjust both rear seat height (seat elevating feature) and backrest angle (backrest recline feature), with a base weight of less than 25 lbs. [13]. This wheelchair provides approximately 25cm of rear seat height adjustment range, along with approximately -5 to +25 degrees of backrest angle recline adjustment [13]. The objective of this study was to investigate and characterize the use of the two dynamic seating functions available on the Elevation^™^ wheelchair during normal everyday usage in the community. A descriptive analysis of seat and backrest adjustments is presented, including frequency of use and duration spent at specific positions throughout daily use. Comparisons between distinct patterns of use by specific subject groupings are also described. In addition, based on anecdotal use reports, it was hypothesized that participants would more frequently use rear seat height adjustments compared to backrest adjustments.

## Methods

### Study design and participants

Cross sectional methods were used for this study. A convenience sample consisting of purposively selected participants were recruited from the lower mainland of Vancouver, British Columbia (BC). The inclusion criteria included individuals who: (1) used the Elevation^™^ wheelchair (PDG Mobility, Vancouver, BC) daily; (2) were at least 19 years old; and (3) were living in the community. The exclusion criteria included individuals who: (1) did not fulfill the inclusion criteria; (2) could not speak and/or write English; (3) were acutely ill; (4) could not provide their own consent; and (5) were living in nursing homes, residential care facilities or long term care facilities. A total of eight participants were recruited for the study. Co-investigators recruited all participants by providing a letter of contact to the manufacturer of the Elevation^™^ wheelchair (formerly Instinct Mobility Inc., Vancouver, BC) who then distributed them to their customers of the Elevation^™^ wheelchair. Ethical approval for this study was obtained through the University of British Columbia Behavioural Research Ethics Board and the Vancouver Coastal Health Authority before the study commenced.

### Data logger instrumentation

**Fig 1:**
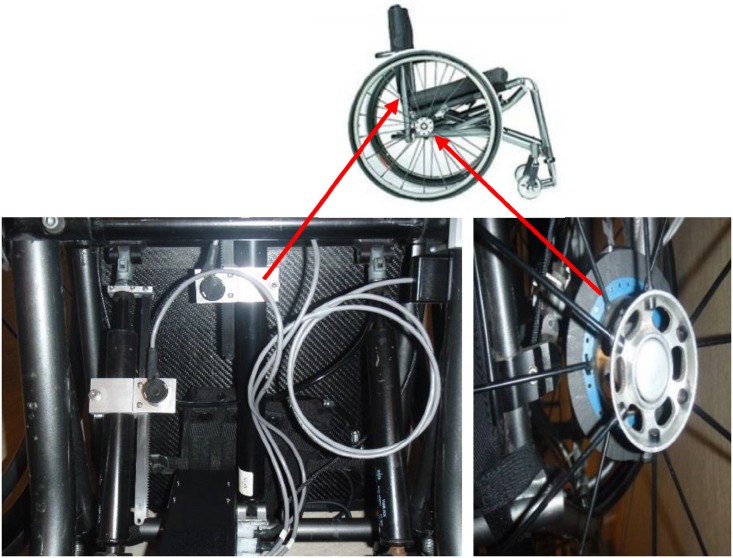
Wheelchair data logger instrumentation showing potentiometers used to determine rear seat height and backrest recline (left) and encoder disc used to measure odometry (right).

Data related to dynamic seating usage, seat occupancy, and odometry were collected using a customized data-logging device, developed at the British Columbia Institute of Technology (BCIT). The data logger consisted of two SparkFun Logomatic v2 dataloggers (SparkFun Electronics, Niwot, CO) coupled to an external battery pack. Two potentiometers were attached to the wheelchair seat back, one each for seat elevation and backrest recline. A chair sensor pad (SMART Caregiver Corporation, Petaluma CA) was installed underneath the seat cushion to measure seat occupancy. A custom optical encoder disc was attached to the wheel to measure wheelchair odometry. All sensors were attached to the data logging device which was secured temporarily to the wheelchair frame under the seat using a plastic snap-on clamp system. See Fig 1 for details. The data logger sampled the potentiometers and occupancy sensor at a rate of 4 Hz, with the raw signal smoothed with a running average of 5 samples. Odometry data was a 250 Hz digital square wave output directly to the data logger, with each square wave corresponding to one division of the optical encoder disk, which consisted of 60 divisions per revolution of the wheel. The data logging system was developed under BCIT’s ISO 9001 Quality System and included validation of the sensors and entire data recording system. The system was capable of collecting up to two weeks of data on a secured digital memory card. After the data collection period, data was downloaded with a memory card reader.

### Study protocol

Individuals who responded to the initial letter of contact were emailed an informed consent form, and two separate meetings were scheduled with each participant. The meetings were held at the participant's’ home, or at a mutually convenient location in the community.

At the start of the first meeting, written informed consent was obtained from the participant. Participants were fully informed about the purpose of the study and the data that was being collected. They were instructed to continue with their normal activities and routines throughout the data collection period, despite chair instrumentation. Next, a demographic survey and the Barthel Index (BI) [14] were administered, and the data-logging device, sensors, and battery were installed onto the participant’s wheelchair. They were given verbal instructions to change the external battery pack once a day and to charge the second battery pack when not in use. A time log sheet was provided for each participant to record the time they attached a charged battery to the data logger. A brief calibration routine was also performed, whereby the subject placed the wheelchair seat into 3 different rear seat heights and 3 different backrest positions. The attending researcher would measure the rear seat height and backrest angle in these positions, and record the results. This data would later be used to calibrate the raw data logger signal to clinically-relevant results of rear seat height and backrest angle. The first meeting lasted one hour.

The second meeting was held nine days after the first meeting to ensure that a full week of data was collected, and lasted two hours. During the second meeting, the data-logging device, battery, and sensors were removed from the wheelchair. The Functioning Everyday with a Wheelchair (FEW) [15, 16] questionnaire, was also administered.

### Demographics and survey instruments

A demographic questionnaire was used to gather participant information and describe the study sample. The demographic survey (created by our team) was used to collect background information from each participant: age, sex, primary diagnosis accounting for wheelchair use, number of years using the Elevation^™^ wheelchair, employment status, highest level of education completed, family support at home, and marital status. The self-report version of the BI was used to describe functional independence with activities of daily living (ADL); higher scores on the BI indicate greater functional independence with ADL (maximum score = 100) [14]. The FEW was used to describe perceived user function related to the Elevation^™^ wheelchair use. The FEW is a self-report questionnaire that probes users’ perceptions of wheelchair functionality related to 10 basic wheelchair uses (e.g. reaching and carrying out tasks at different surface heights, transferring, and carrying out personal care tasks). Higher FEW scores indicate a better perceived match between the wheelchair and user needs (maximum score is 60) [15].

### Data analysis

Only seven of the nine days of data collection were analyzed because the first and last days involved interactions between participants and researchers, which disrupted participants’ normal routines. Raw data stored on the secure digital memory card was downloaded onto a personal computer and analyzed using MATLAB (Mathworks Inc., Natick, MA). Using the algorithms described below, the raw data was transformed into daily seated occupancy, seating positions and adjustment frequencies (seat elevation, and backrest angle), and distance traveled.

Seat occupancy was defined as any time spent in the wheelchair in which data was recorded as occupied pressure for greater than 60 seconds. The total hours each participant spent in their wheelchair each day was calculated.

Rear seat height adjustments were defined as any rear seat elevation change greater or equal to 1.5 cm that lasted for at least five seconds. The number of times each participant accessed their seat elevation feature was calculated for each of the seven days. Seat elevation adjustment frequency/hour was calculated by using the total hours of seat occupancy from each day, and was calculated to provide a more accurate representation of adjustment usage because not every participant spent the same amount of time in their wheelchair every day.

Backrest angle adjustments were defined as any backrest angle change greater or equal to 2 degrees that lasted for at least 20 seconds, similar to others’ work [17]. A dwell time of 20 seconds was chosen in order to eliminate confounding temporary readings, e.g. during wheeling, or when a subject briefly leaned rearwards during weight shifts or otherwise. The number of times each participant adjusted their backrest angle was calculated for each of the seven days. Backrest angle adjustment frequency/hour was calculated by using the total hours of seat occupancy from each day.

Distance traveled was expressed in meters for each of the seven days. The average and median distance traveled was calculated and expressed as meters/day for each participant.

Descriptive statistics, including means, standard deviations, and frequencies were calculated, as well as medians and ranges. Nonparametric statistics were used to test hypothesized median differences due to relatively small sample size and skewed distributions in the data. A one-tailed Wilcoxon test for paired data was used to compare the frequency of seat vs. backrest adjustments, with confidence intervals based on Walsh Averages. A one-tailed Mann–Whitney U-test for unpaired data was used to compare habitual vs. varying rear seat height adjusters (see Results).

## Results

### Sample characteristics

Sample characteristics of participants, BI, and FEW scores are summarized in Table 1. A total of eight participants completed the study, including seven males and one female. The median age (and range) was 40.5 (35–52) years old. Six participants had traumatic SCI ranging from T12 to C5, one participant had a non-traumatic SCI (spinal cancer), and one participant had cerebral palsy. Two participants reported that that they were able to ambulate with the use of a walking aid. The majority (7 out of 8) of the participants had a BI of 70 or over, and were living independently in the community. A single participant had a BI score of 40 and required partial care-giver support with self-care activities. This single participant was a C5 tetraplegic, and used the Elevation^™^ wheelchair in a unique way compared to the other participants, partly facilitated by custom modifications (e.g. thumb loops to activate adjustment features, a limit strap to prevent seat elevations higher than level sitting, and a custom centre-mounted anti-tip caster fork to allow leaning back for comfort). The group median FEW score was 54 (range = 47 to 60), indicating a high perceived match of the Elevation^™^ wheelchair with the user. It was clear from observations and feedback that each participant was fully capable of understanding how to physically make dynamic “on the fly” adjustments to their wheelchair. All participants had similar *ranges* of adjustment capability of both rear seat height and backrest angle, with the exception of Subject 3 as described above. However with regards to rear seat height, participants may have had different minimum rear seat heights from which their range would follow upwards from; this was a factory or dealer adjustment set prior to use, and based on client and therapist input.

**Table 1:** Participants sample characteristics.

Sub. #	Age	Sex	Diagnoses	Years using EW/C	Employment	Education	Family support at home	Marital Status	Barthel Index	FEW Score
**1**	46	M	SCI T8	4	Part Time	University	None	Single	80	54
**2**	50	F	SCI T7/T8	1	Full Time	Post Grad.	None	Single	80	54
**3**	36	M	SCI C5	2	Part Time	College	None	N/A	40	57
**4**	35	M	Cancer of spine	2	Full Time	High school	Parents	Single	100	60
**5**	52	M	Cerebral Palsy	3	Full Time	College	Spouse & 3 children	Married	95	47
**6**	41	M	SCI T12	3.5	Unemployed	College	None	Married	80	50
**7**	40	M	SCI T10	4	Unemployed	High school	Spouse	Married	80	48
**8**	40	M	SCI T12	2.5	Unemployed	High School	Son	Single	70	59

M = male, F = female; SCI = spinal cord injury, T = thoracic, C = cervical; EW/C = Elevation^™^ wheelchair

### Description of Elevation^™^ wheelchair use in the community

Seat elevation and backrest angle adjustment frequencies, seat occupancy, and distance traveled per day were tabulated for each participant (see Table 2). Due to technical problems in the field, some data were not collected: for Participants 4 and 5 only six days of data were recorded and for Participants 2 and 7 no odometry data was recorded due to incorrect installation of the equipment.

**Table 2:** Data logging results, both single subject and grouped data, for: Odometry (meters/day), seat occupancy (hours/day), and dynamic seating access (frequency/hour).

Participant	Mean distance travelled/Day	Mean distance/ hour occupied	Mean hours of seat occupancy/day	Mean seat elevation changes/hour	Mean backrest angle changes/hour
	(# of days collected)	(# of days collected)	(# of days collected)	(# of days collected)	(# of days collected)
1	2416	228	10.6	1.66	0.013
	(7)	(7)	(7)	(7)	(7)
2	-		11.8	2.13	0.012
	(0)	(0)	(7)	(7)	(7)
3	1240	87	14.2	0.56	1.77
	(7)	(7)	(7)	(7)	(7)
4	3484	425	8.2	0.71	0.12
	(6)	(6)	(6)	(6)	(6)
5	650	83	7.8	0.90	0.79
	(6)	(6)	(6)	(6)	(6)
6	1810	165	11.0	4.04	0.14
	(7)	(7)	(7)	(7)	(7)
7	-		11.1	0.55	0.35
	(0)	(0)	(7)	(7)	(7)
8	510	59	8.7	0.67	0.099
	(7)	(7)	(7)	(7)	(7)
Mean	1685	174	10.4	1.40	0.41
(± SD)	(463)	(56)	(0.8)	(0.43)	(0.21)
Median	1525	126	10.8	0.81	0.13
(Range)	(510–3484)	(59–425)	(7.8–14.2)	(0.55–4.04)	(0.01–1.78)

Participants spent a median time in their wheelchairs (i.e. occupied sitting) of 10.8 hours (range = 7.8 to 14.2 hours). All participants were active manual wheelchair users, with a median distance of travel of 1525 m/day (range = 510 to 3480 m/day). One participant (Participant 4—a participant with a SCI due to cancer of the spine) reported that he rarely used his wheelchair within his home, instead opting to stand, walk with wall/furniture support, kneel, and crawl. Similarly, Participant 5 (a participant with CP) would often ambulate within his home using various supports.

**Fig 2:**
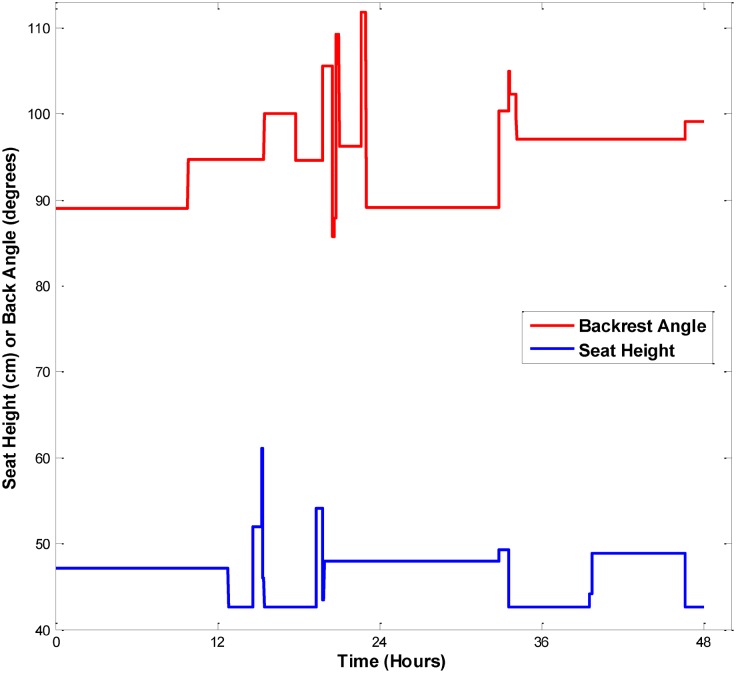
Pattern (by the minute) of daily seat height and backrest angle position over two days for a single participant.

The number of rear seat height activations and backrest angle activations had median (and range) values of 0.81 (0.55–4.04) and 0.13 (0.01–1.78), activations per hour respectively, across all participants (Table 2). Both feature activations per hour of use had positive skew values, attesting to these behaviours trending to lower typical hourly usage frequencies. The hypothesis of whether participants would more frequently use rear seat height adjustments compared to backrest adjustments was tested, using median values as appropriate for this small sample size and skewed data. Rear seat height adjustments were accessed significantly more often than the backrest angle adjustments (p = 0.039; median difference estimate of 0.90, with a 95% confidence interval of the median difference of -0.32 to 2.24). A representative sample of rear seat height and backrest adjustment activity for Participant 5 is shown in Fig 2.

The backrest adjustment feature was, with the exception of a single participant, far less used than the seat height adjustment feature. Two participants in fact only adjusted their backrest once during the entire data collection period; although, half the participants did change their back angle more than once per day on average. If we subdivide the participants into those using their backrest angle adjustment feature less than once per day (infrequent users), and those using the feature more than once per day (frequent users), we find that frequent users activated their backrests about 10 times more often than infrequent users, with medians (and ranges) of 0.57 (0.14–1.8) and 0.056 (0.012–0.12) activations per hour, respectively.

### Characteristics of “on the fly” rear seat height adjustments

The observation of the relatively minimal usage of the backrest angle adjustment feature led to an analytical focus on the patterns of rear seat height adjustment, of which there seemed to be interesting differences among participants upon inspection. First, there was a considerable difference in the typical rear seat heights at which individual participants spent their time (see Table 3). For example, Participant 5, with the highest daily rear seat height (calculated over the entire data collection period, averaged by the minute), sat at mean of 46.7 ± 3.6 cm and median of 47.2 cm. In contrast, Participant 7, with the lowest daily rear seat height, sat at a mean of 34.1 ± 2.7 cm and median of 33.0 cm.

**Table 3:** Mean and median seat heights for each subject, calculated over the entire data collection period, by the minute.

Participant	Mean seat height (cm)	Median seat height (cm)
	(SD)	(Range)
1	40.0	38.1
	(2.7)	(37.8–60.6)
2	43.0	42.1
	(2.4)	(41.9–53.0)
3	38.4	37.6
	(3.0)	(33.0–47.3)
4	39.7	34.4
	(6.1)	(34.1–48.2)
5	46.7	47.2
	(3.6)	(42.6–61.1)
6	43.5	42.9
	(3.6)	(37.0–62.9)
7	34.1	33.0
	(2.7)	(32.9–50.0)
8	39.0	39.0
	(0.6)	(38.9–60.2)

**Fig 3:**
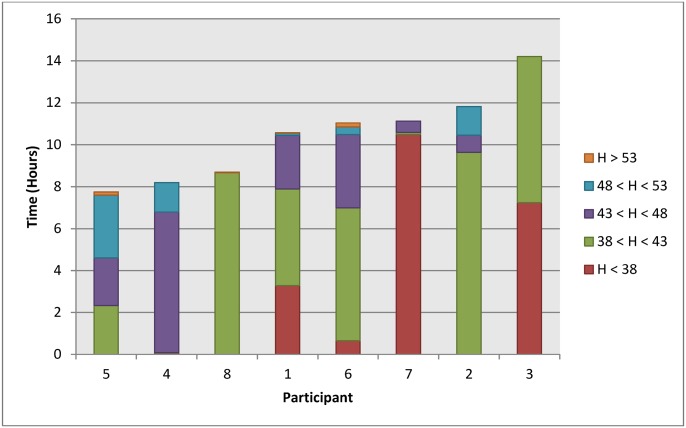
Pattern of daily mean occupied sitting time in the wheelchair for each participant, characterized by seat height ranges, in centimeters. H = height (cm).

**Fig 4:**
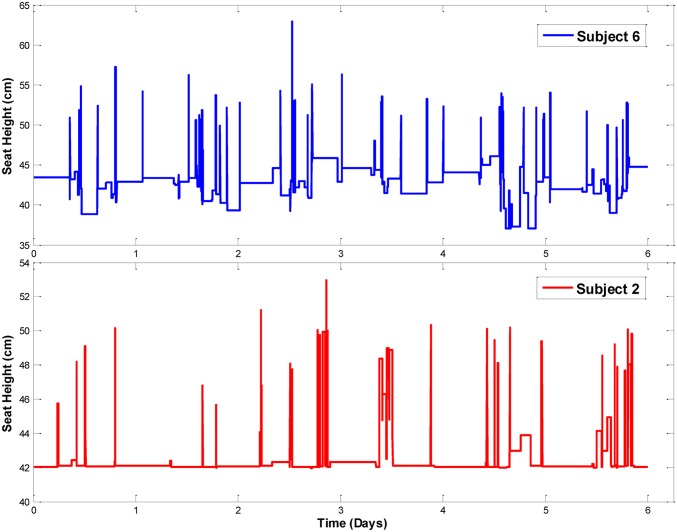
Habitual (bottom) and varying (top) seat height data (by the minute) over 6 days of data collection.

In order to visualize a more complete description of the diversity in rear seat height ranges that each participant used, we calculated a breakdown of the overall daily time each participant spent at five different heights bins within the entire height range available to all participants. This is graphically depicted in Fig 3. A distinct pattern of typical daily use by different participants was observed. One sub-group of 4 participants habitually spent more than 80% of their time in a single bin of rear seat heights (Table 4). The other sub-group had a more varying rear seat height use, spending no more than 58% in a single rear seat height bin. A comparison between the two sub-groups revealed that the habitual and varying groups had median (and range) values of 88% (82–100) and 47% (39–58), respectively, of the amount of time spent in their most frequented rear seat height bin). Of the 4 participants in the habitual sub-group, there were three different rear seat heights for their most usual positions: Participant 7 usually sat at the lowest height bin; Participants 2 and 8 usually sat at the second lowest bin; and Participant 4 sat at the middle of the five bins. We also hypothesized that the varying sub-group had changed their rear seat height more frequently than the habitual sub-group. This was the trend, with medians (and ranges) of 1.28 (0.56–4.0) and 0.69 (0.55–2.1) activations per hour, respectively, although the result was not statistically significant. Several days of rear seat height activity for both a habitual and varying user is shown in Fig 4 for comparison purposes.

**Table 4:** Habitual and varying seat height use.

Habitual seat heights			Varying seat heights		
Participant	Seat changes/hour	% time spent in most common position	Participant	Seat changes/hour	% time spent in most common position
2	2.13	81.7	1	1.66	43.6
4	0.71	81.8	3	0.56	51.0
7	0.55	94.4	5	0.9	38.6
8	0.67	99.7	6	4.04	57.5
Mean	1.01	89.4		1.79	47.7
(SD)	(0.37)	(4.5)		(0.78)	(4.1)
Median	0.69	88.1		1.28	47.3
(Range)	(0.55–2.13)	(81.7–99.7)		(0.56–4.04)	(38.6–57.5)

## Discussion

Dynamic seating features on PWC and conventional MWC are not a new concept; however they have not been available on ultralight manual wheelchairs until the recent development of the Elevation^™^ wheelchair. This new form of assistive technology is unique to wheelchair consumers because it allows users to self-adjust “on the fly” their rear seat height and backrest angle on an ultralight manual wheelchair throughout normal daily activities. This pilot study aimed to investigate and characterize the use of the backrest and rear seat height adjustment functions of the Elevation^™^ wheelchair during everyday usage in the community. While others have studied dynamic seating in other classes of wheelchair [17–19], this study is the first to investigate the use of these features in an ultralight wheelchair.

Our study findings indicate that the typical rear seat height used by participants varied considerably from user to user. The dynamic rear seat height usage of the participants with the highest and lowest mean rear seat heights (i.e. Participants 5 and 7 with mean rear seat heights of 46.7 cm and 34.1 cm respectively) provide an interesting illustrative example. These two participants had very different disabilities and wheelchair configurations. Participant 5, who sat the highest on average, was an ambulatory man with cerebral palsy who had the function to stabilize and effectively use the chair at these higher heights. In order for the wheelchair to function at these higher ranges, it was configured by the therapist and dealer with higher minimum rear seat height, a configurable option for this product [20]. In contrast, Participant 7 was a man with a T10 level SCI. His wheelchair was configured with the lowest possible rear seat height of 32.9 cm from the floor. These results may indicate that users, when given the option of adjusting rear seat height, may naturally adapt usage to best suit their disability and functional abilities. The importance of tailoring seating position and appropriate trunk support to the variability of wheelchair users’ needs has been noted in the literature [21, 22]. Given that most manual wheelchairs are configured to a fixed seat position, our findings may provide additional support for the use of dynamic seating to optimize functional ability.

In addition to the variability noted with rear seat height positions, daily adjustment patterns were also found to be variable. Our study revealed two distinct types of rear seat height adjustment patterns amongst our participants: those who spent most of their seated time at a “habitual” rear seat height, and those whose rear seat height patterns were more varied. These findings mirror the findings of Sonenblum et al. [17] who explored adjustability patterns of tilt-in-space systems amongst PWC users. They identified two distinct groups of users: those who spent at least 80% of their seated time in a single position, and those who had no meaningful typical position (i.e spent <80% of time at any single position). Using this definition, our study participants demonstrated similar rear seat height adjustment patterns, with half of our study group (N = 4) using habitual rear seat heights and half (N = 4) using more varying rear seat heights.

Within these sub-groups, it is interesting to note that there was considerable variation in the most usual rear seat height for users in the habitual group—i.e. of the 4 users in this category, there were 3 different habitual rear seat heights. The findings also indicate that the varying sub-group changed their rear seat height more frequently than the habitual sub-group. While analysis of our findings did not show any significant correlation with feature usage and years of wheelchair use, Sonenblum’s study with PWC users showed that the number of years in a wheelchair was negatively associated with tilt frequency [17]. These results open up a number of questions for future studies. For example, are rear seat height adjustment patterns related to the variability of the tasks participants do in a day (i.e. do users in the habitual sub-group conduct fewer different tasks throughout the day)? Could independent wheelchair settings such as rear axle position or cushion type affect seat height adjustment behaviours? (Neither of these variables were recorded in this study.) Are there specific reasons why a limited number of rear seat heights work best for these habitual users (e.g. stability, positioning, usability)? And what impact would training have on adjustment frequency?

The findings confirmed our hypothesis that backrest adjustment would be used much less than the rear seat height adjustment. In fact, only one participant adjusted the backrest more than once per hour. We were able to define two sub-groups of backrest users: frequent and infrequent adjusters. Frequent adjusters were found to have adjusted the backrest feature ten times more than the infrequent adjusters, indicating that one sub-group may have “bought into” the concept of backrest adjustment, whereas the other sub-group may not have. It is also possible that, as with the use of the rear seat height adjustability feature, backrest adjustability usage may have been influenced by seat cushion and/or rear axle position. These configurations can impact both the wheelchair user’s comfort and stability and could potentially have influenced how often the backrest feature is accessed. With regards to rear axle position, this pre-set wheelchair configuration parameter is independent of “on the fly” seating adjustments, allowing user-preferred “tippiness” to be optimized. Users with particularly tippy wheelchairs may have used their backrest adjustment less often due to stability concerns. While these effects were not considered in this study, further investigation is warranted.

It is worth noting that even amongst the frequent backrest adjusters, the usage of this feature was still low compared to rear seat height adjustments. This finding is surprising, given that the backrest has been described as a critical piece of wheelchair componentry due to the importance of seating position and appropriate trunk support for wheelchair users [21, 23]. As individual users have individual preferences for their postures during daily activities [21], adjustable backrests have the potential to provide users with custom positioning tailored to their unique circumstances. Specifically, adjustable backrests can facilitate a range of activities (e.g. dressing, napping, self-catheterization, leg stretches, and exercises [18, 19, 21]) as well as address certain medical needs (e.g spasticity, pressure management, comfort, contractures, and edema) [10]. Adjustable backrests may also impact manual wheelchair users wheeling on hills and ramps, providing users with a greater sense of stability and safety, minimizing injuries, and facilitating wheeling [21]. When going uphill, the backrest can be adjusted forward to provide support to users leaning into the slope. Similarly, the backrest can be adjusted to a greater recline to provide the user with trunk support and forward wheeling stability when going down a hill [21]; this simple “on the fly” adjustment may obviate the need to perform a “wheelie” when traveling down steep slopes, thus enabling people with less function to perform this type of wheeling and potentially increasing the safety of such community wheeling in general.

Although each participant in our study had full knowledge of how to adjust their backrest and rear seat height position, we did not gather any data about whether they were informed of specific applications where these adjustments would be beneficial. Thus it was unclear to the extent to which they understood the functional implications of making adjustments or the appropriate situations to use them. Elevation Wheelchair ^™^ owners receive a basic user manual at the time of purchase which focuses primarily on setting up and maintaining the wheelchair, as well as basic instruction of how to physically use the seat adjustment controls. No information is provided about the nuances of usage or appropriate applications of usage. Given the potential benefits of these adjustability features, this may point to the need for training initiatives to help users better understand when to use them.

Findings from our study indicate that participants on average spent 10.4 +/- 0.8 hrs /day occupying their wheelchairs. Our results are slightly higher than those reported in by Yang (mean = 9.2 hr/day) [24] and Tolerico (8.3 ±3.3 hrs/day) [25], who also looked at occupancy amongst manual wheelchair users. (Note- our study and Yang’s results considered total occupancy time including time sitting stationary in a wheelchair, whereas Tolerico’s findings considered only active time when users traveled >50 m/ hour). Studies looking at occupancy amongst PWC users report similar or slightly higher occupancy rates amongst this population (likely since these users only perform a limited number of transfers in and out of their wheelchairs each day [19]). Reported mean occupancy in PWC has included 10.8+/- 2.9 hrs/day [26], 11.7+/- 3.7 [17], and 11.8 ± 3.4 hours a day [19]. It is evident from these studies that both MWC and PWC users spend considerable time sitting in their wheelchairs. In the able bodied population, prolonged periods of sitting have been associated with negative health outcomes [27, 28] and people without disabilities typically require frequent changes of position while sitting. The implications of prolonged periods of sitting may be even more serious for people with disabilities who may not be able to readily change their position. In a study involving PWC users, 59% of users felt their pain was influenced by their wheelchairs and 30% reported pain or discomfort aggravated by sitting [22]. Frequent repositioning in the wheelchair has been strongly recommended [21].

While this study provides insight into frequency of use of the dynamic “on the fly” adjustment features in the Elevation^™^ wheelchair, the study did not consider why these features were being used. The literature indicates that use of dynamic seating features in PWC has the potential to enable activities of daily living, independence, and participation in life areas such as productivity (work/school), communication, parenting, social life, self-care, meal preparation, and shopping [9, 10]. Both shopping and working can present some unique challenges during daily manual wheelchair use [9] as many objects and surfaces (e.g. workbenches, shelves, and cash register stands) are difficult for users of standard ultralight manual wheelchairs to reach. It is reasonable to assume that the Elevation^™^ wheelchair’s dynamic seating features were used to support these activities, however qualitative studies exploring how these features are used are required to confirm this and provide a greater depth of understanding of the context of usage.

As inadequate seating systems or ineffective usage of such systems can lead to increased costs, pressure sores, pain, fatigue, and unnecessary limitations to ADL [9, 29], it is important that future studies be conducted to answer the many questions raised from this work. These findings have the potential to impact both clinical practice and wheelchair design. Prescribers of the technology may use this information to better match users with wheelchairs that best suit their unique situations (e.g. based on their disability, functional abilities, and participation goals). Clinicians may use this information to better develop and implement training programs to teach users about feature usage. Currently, very little training is provided to Elevation^™^ wheelchair users to guide them on optimal rear seat height and backrest positioning. Skills training has been shown to increase wheeling proficiency and confidence amongst manual wheelchair users [30–32]. For PWC users, it has been suggested that training and education may promote increased use of dynamic functions [17]. It is anticipated that specific training for Elevation^™^ wheelchair users would also lead to more effective dynamic feature use, resulting in improved function, participation, and quality of life. Finally, a better understanding of dynamic feature usage may provide insight for future wheelchair designs.

### Study limitations

Due to the sample size, the objective wheelchair data was not normally distributed, and the results cannot be generalized beyond the current sample. The sample lacked variability in functional independence with ADLs, and primary diagnoses that accounted for participants’ wheelchair use (i.e. the majority had mid-level SCI), which reduced the heterogeneity of the sample.

Other limitations should also be noted. Firstly, knowledge of the expertise levels of participants (i.e. their nuanced understanding of the functional implications of making adjustments or appropriate usage situations) was not evaluated. Secondly, the presence of upper limb pain was not recorded and did not form part of our inclusion/exclusion criteria. Thirdly, seat cushion type and rear axle position were not recorded and it is acknowledged that these personalized wheelchair components may have had an impact on a users’ stability and/or comfort. It is possible that all these factors may have influenced seating adjustment patterns. Further, the data collection period was limited which may not have reliably captured typical usage patterns. Also, some data were missing due to technical difficulties in the field. Finally, participant behaviours may have been impacted by the Hawthorne effect [33]—i.e. they may have modified their behaviours due to the fact that they were aware that they were being monitored.

### Future research

This study provides preliminary evidence regarding the characteristic usage of dynamic seating features on ultralight manual wheelchairs, collected with the use of data loggers. However, it remains unanswered as to exactly how individuals use their dynamic seating features to assist with mobility related activities, ADLs, and participation. Qualitative research to study context (e.g. how, why, when dynamic seating features are used) is recommended, which will also provide greater insight into the benefits and limitations of this technology. The inclusion of more disability groups is also suggested to generalize the findings to a wider population. Once the Elevation^™^ wheelchair and other products such as the “Lightweight, Durable, Adjustable Composite Backrest Mounting” [21] are more widespread in the market, future cohort studies could also compare between groups to determine how dynamic seating impacts activity and participation outcomes. For instance, one study could compare ultralight manual wheelchair users with and without dynamic seating; another intervention study could investigate users pre- and post-delivery of an ultralight manual wheelchair with dynamic “on the fly” seating features.

## Conclusions

This exploratory study provided insight into the usage of an ultralight manual wheelchair with “on the fly” seating adjustment features. Analysis of rear seat height and backrest adjustment data revealed considerable variability in the positions used by participants. Two sub-groups of users were identified: those who sat habitually at a single typical rear seat height, and those who varied their rear seat height more continuously. Findings also indicate that participants used the rear seat height adjustment feature significantly more than the backrest adjustment feature. This obvious contrast in feature use may indicate that new users of this class of wheelchair may benefit from specific training. While the small sample size and exploratory nature of this study limit the generalizability of our results, our findings offer a first look at how active wheelchairs users are using a new class of ultralight wheelchair with “on the fly” seating adjustments. Further studies exploring the daily use context of feature adjustment are recommended to better understand the impact of dynamic “on the fly” seating adjustments on activity, participation and quality of life of active ultralight wheelchair users in the community.
